# Supplementary material for: Descriptive epidemiology of physical activity energy expenditure in UK adults (The Fenland study)
Source: Int J Behav Nutr Phys Act. 2019 Dec 9;16:126. doi: 10.1186/s12966-019-0882-6 (PMC6902569; doi:10.1186/s12966-019-0882-6)
Supplement: Supplementary file 2 — Additional file 2: Figure S1. Sex stratified time (minutes per day) across the full spectrum of physical activity intensities in the Fenland cohort. Men accumulated higher levels of very vigorous physical activity than women, although both sexes accumulated relatively little activity over 5 METs. Box plots represent medians and interquartile ranges. [file 12966_2019_882_MOESM2_ESM.pptx]

## Slide 1
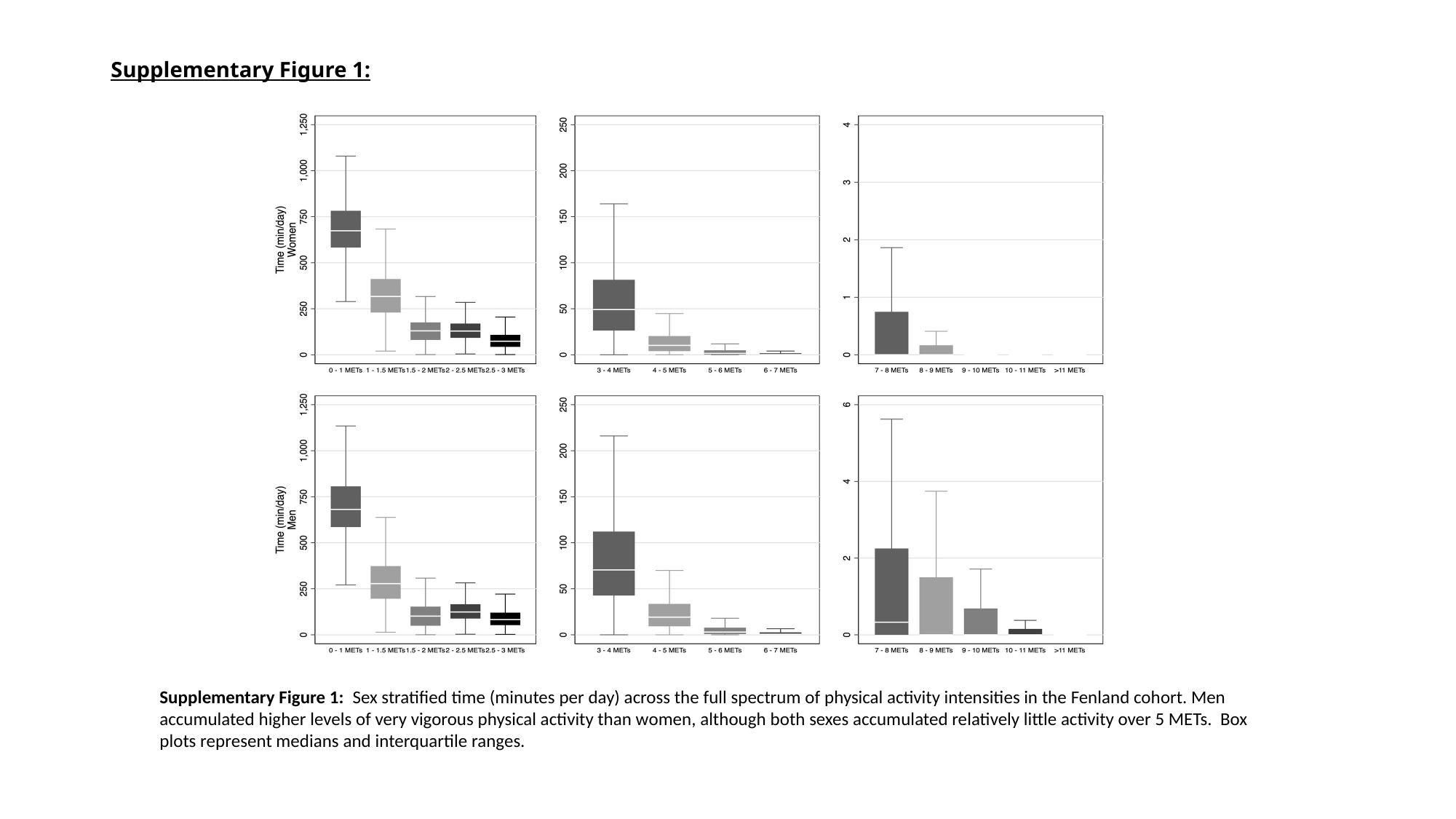

Supplementary Figure 1:
Supplementary Figure 1: Sex stratified time (minutes per day) across the full spectrum of physical activity intensities in the Fenland cohort. Men accumulated higher levels of very vigorous physical activity than women, although both sexes accumulated relatively little activity over 5 METs. Box plots represent medians and interquartile ranges.
